# Global burden of ovarian cancer attributable to high body mass index among women of childbearing age from 1990 to 2021 and projections to 2050: a systematic analysis for the global burden of disease study 2021

**DOI:** 10.3389/fonc.2025.1695717

**Published:** 2025-12-18

**Authors:** Hongxi Chen, Zidan Lin, Jing Chen, Shanyang He

**Affiliations:** 1 Guangdong Cardiovascular Institute, Guangdong Provincial People’s Hospital, Guangdong Academy of Medical Sciences, Guangzhou, China; 2 Department of Gynaecology and Obstetrics, Guangdong Provincial People’s Hospital (Guangdong Academy of Medical Sciences), Southern Medical University, Guangzhou, China

**Keywords:** BMI, disease burden, GBD 2021, ovarian cancer, women of childbearing age (WCBA)

## Abstract

**Background:**

Ovarian cancer (OC) remains a lethal gynecologic cancer marked by substantially reduced 5-year survival probabilities. Elevated BMI (≥25 kg/m²) constitutes a progressively recognized OC risk determinant mediated by chronic inflammatory states and metabolic pathway perturbations. Leveraging GBD 2021 repositories, this investigation quantified high BMI-attributable ovarian cancer burden trajectories among reproductive-age women (WCBA, 15–49 years) worldwide.

**Methods:**

Leveraging GBD 2021 repositories, we evaluated worldwide OC burden mediated by elevated BMI across women of childbearing age (WCBA) during 1990-2021. Evaluated metrics comprised deaths, DALYs, YLDs, YLLs, and ASR (EAPC-based trend analysis). ARIMA and Exponential Smoothing models generated 2050 burden projections.

**Results:**

From 1990 to 2021, the global burden of OC attributable to high BMI increased significantly. In 2021, there were 17,344 deaths (95% UI: 4,141–30,810) and 477,248 DALYs (95% UI: 113,449–840,002) among WCBA. The ASMR rose from 0.32 (95% UI: 0.07–0.61) to 0.38 (95% UI: 0.09–0.67) per 100,000 (EAPC: 1.03, 95% CI: 0.85–1.21). The ASDR increased from 8.72 (95% UI: 1.78–16.41) to 10.56 (95% UI: 2.50–18.57) per 100,000 (EAPC: 1.09, 95% CI: 0.93–1.25). The burden peaked in the 45–49 age group, with 995 deaths and 44,223 DALYs. High SDI regions had the highest ASMR (0.57, 95% UI: 0.14–1.01) and ASDR (15.13, 95% UI: 3.79–26.82). Projections indicate a continued increase in the OC burden attributable to high BMI by 2050, with the ASMR reaching 0.43 (95% HDI: 0.40–0.46) and the ASDR reaching 12.28 (95% HDI: 11.58–12.98).

**Conclusion:**

This study highlights the escalating global burden of OC attributable to high BMI among WCBA, particularly in high SDI regions. This investigation delineates progressively intensifying worldwide OC burden mediated by elevated BMI in WCBA, disproportionately affecting high SDI territories.

## Introduction

Among gynecological malignancies worldwide, ovarian cancer (OC) maintains exceptionally poor survival outcomes, with 5-year survival rates substantially below those of comparable female reproductive tract cancers (1, 2). The elevated mortality associated with OC is predominantly attributable to advanced-stage diagnosis, with current epidemiological data indicating a 5-year survival rate of merely 47% (3). However, accumulating empirical evidence underscores the role of BMI modulation (body mass index) in ovarian carcinogenesis (4). The standard BMI metric derives from the quotient of weight (kg) and height squared (m²), where values >25 kg/m² indicate clinically significant elevation according to WHO classification (5). Mechanistic investigations have revealed that genetic predisposition (e.g., BRCA1/2 mutations) and endocrine dysregulation (particularly estrogen-progesterone axis alterations) contribute to OC pathogenesis (6). Specifically, OC pathogenesis and disease progression are substantially influenced by persistent inflammatory states resulting from obesity, manifesting as insulin pathway dysregulation and adipokine homeostasis impairment (7, 8). At the same time, BMI is an indirect proxy of adiposity and metabolic risk and does not capture body fat distribution or lean mass; recent work demonstrates that body-composition phenotypes and visceral adiposity can be more strongly associated with cancer outcomes, including ovarian cancer, than BMI alone (34, 35). Moreover, meta-analytic evidence indicates that the relationship between anthropometric factors and OC risk differs across histologic subtypes and menopausal status, underscoring the biological heterogeneity underlying adiposity–OC associations (33). To situate this work within the broader GBD 2021 landscape of female cancers, we note that breast cancer drives the highest incidence globally, cervical cancer patterns vary widely by screening and HPV vaccination coverage, uterine cancer has risen in tandem with adiposity, and ovarian cancer remains the most lethal among the four major female cancers; these context-setting trends are summarized in recent GBD-based syntheses (29).

Although these relationships are documented, methodologically robust investigations quantifying worldwide OC burden specifically linked to high BMI levels in 15–49 year old women remain scarce (9). While prior investigations have predominantly focused on regional populations or particular histopathological subtypes (e.g., serous epithelial OC), they have generally omitted the systematic integration of age-standardized mortality rates (ASMR), disability-adjusted life years (DALYs), and longitudinal projections (10). Moreover, variations among Socio-Demographic Index (SDI) quintiles and age-specific groups remain inadequately characterized, hindering the development of targeted preventive strategies (10). In global health and demographic reporting, women of childbearing age (WCBA) are conventionally defined as those aged 15–49 years, a period during which OC carries unique implications for fertility preservation, pregnancy planning, and long-term quality of life despite accounting for a minority of all OC deaths. In addition, the heterogeneity of adiposity–OC associations across histologic subtypes and menopausal categories highlighted by recent meta-analyses has not yet been translated into global, risk-attributable estimates for WCBA (33). In this context, by isolating high BMI–attributable OC burden in WCBA, our estimates are designed to complement all-age GBD 2021 analyses rather than replace them, and they should not be generalized to predominantly postmenopausal disease outside the 15–49-year range (29).

Utilizing the GBD 2021 database, this research quantifies historical trajectories (1990-2021) and models prospective burdens (2050 horizon) of ovarian cancer attributable to elevated BMI, directly targeting identified evidence gaps (11, 12). Systematic assessment of obesity-attributable OC mortality and morbidity burdens provides foundational evidence for developing targeted risk stratification frameworks and equitable resource distribution strategies across heterogeneous populations. By focusing specifically on WCBA (15–49 years), our analysis complements all-age GBD evaluations of gynecologic cancers, providing age-specific, high BMI–attributable estimates that are directly relevant to reproductive health policy and pre-/perimenopausal care (10, 29).

## Materials and methods

### Data source

The GBD 2021 repository (https://ghdx.healthdata.org/gbd-2021) constituted an extensive epidemiological resource quantifying disease burden impacts from 371 pathologies, 88 risk factors, and injuries spanning five Socio-Demographic Index (SDI) categories and 204 nations and territories. The SDI operates on a continuous scale of 0-1, where zero denotes minimal developmental attainment and unity indicates maximal development status. Countries and territories were classified into five groups based on SDI quintiles: high SDI (>0.81), high-middle SDI (0.70-0.81), middle SDI (0.61-0.69), low-middle SDI (0.46-0.60), and low SDI (<0.46) (13). Utilizing data from the Global Burden of Disease (GBD) 2021 Study, we extracted global burden estimates for BMI-attributable OC among women of childbearing age (WCBA) covering the period 1990-2021. These estimates included deaths, disability-adjusted life years (DALYs), years lived with disability (YLDs), years of life lost (YLLs), and their corresponding age-standardized rates (ASR). In this study, women of childbearing age were defined as those aged 15–49 years, consistent with standard GBD age groupings. Within the GBD comparative risk assessment, “high BMI” is treated as a continuous risk factor defined in adults using BMI ≥ 25 kg/m², and ovarian cancer is modelled as a single cause without histologic or menopausal stratification. Our estimates therefore reflect the aggregated high BMI–attributable OC burden across all histologic subtypes occurring in women aged 15–49 years rather than subtype- or menopausal-status–specific effects.

### Statistical analysis

The age-standardized mortality rate (ASMR), age-standardized DALYs rate (ASDR), age-standardized YLDs rate (ASYR) were utilized as indicators to evaluate the disease burden of OC attributable to high BMI. The Estimated Annual Percentage Change (EAPC) was used to analyze trends in ASR over a specified time interval. The formula for EAPC was calculated as:


$$EAPC=(e^{\beta }-1)\times 100\%$$

where β is the slope from the linear regression of the natural logarithm of the ASR on the year (14). Confidence interval (*CI*) of EAPC can be obtained from the linear regression model.

All statistical computations employed R software (v4.1.0). Statistical significance was defined as p<0.05. We computed 95% uncertainty intervals (UIs) by extracting the 2.5th-97.5th percentile range across 1,000 posterior distribution iterations per estimation phase. Projections of the burden attributable to BMI for OC through 2050 were developed employing Autoregressive Integrated Moving Average (ARIMA) and Exponential Smoothing (ES) modeling frameworks. These classical univariate time-series approaches were selected because they are widely applied to annual, non-linear disease trends and provide transparent parameterization and diagnostics. For each outcome (ASMR, ASDR, ASYR and YLLs), we fitted ARIMA and ES models to the 1990–2021 series following standard Box–Jenkins procedures, including visual inspection of temporal patterns, assessment of stationarity, and comparison of candidate model orders using information criteria to guide model selection. Residual diagnostics were examined to ensure that substantial autocorrelation was not present in the final models. For all projections we derived 95% prediction intervals (expressed as highest density intervals, HDIs), which are reported alongside point forecasts in the text. ARIMA and ES yielded qualitatively similar trajectories, providing internal consistency for our projections.

## Results

### The global burden of OC attributable to high BMI from 1990 to 2021

**Figure 1:**
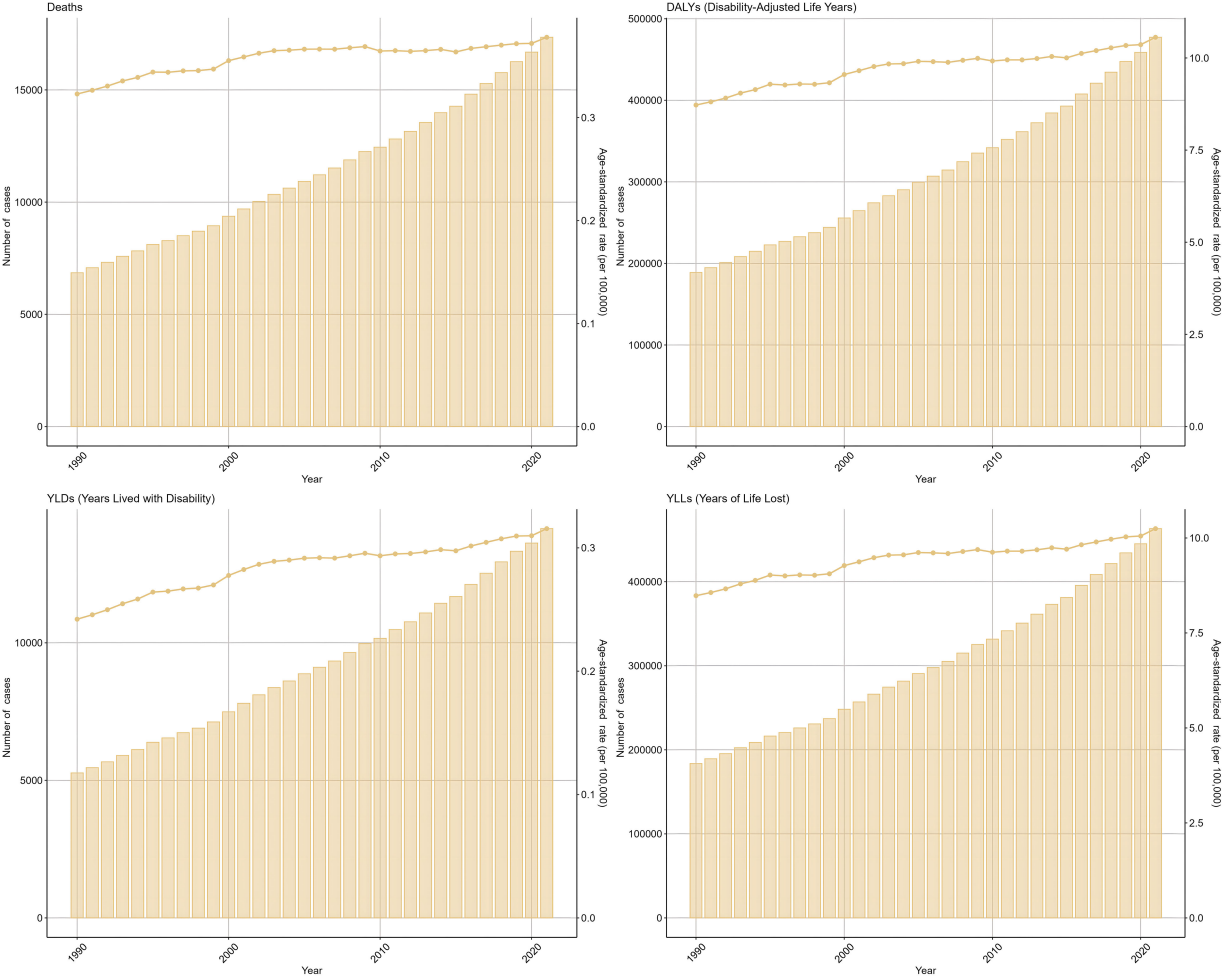
Trends in the global burden of OC attributable to high BMI from 1990 to 2021. Indicators include ASMR, ASDR and ASYR (rates per 100,000) and counts (deaths, DALYs, YLDs, YLLs) as applicable; where shown, uncertainty is 95% uncertainty intervals (UIs).

In 2021, there were 17344 (95% UI: 4141-30810) deaths among WCBA caused by OC attributable to high BMI, increased from 6850 (95% UI: 1422-12864) in 1990. Similarly, the DALYs for OC attributable to high BMI increased from 188874 (95% UI: 38401-355691) in 1990 to 477248 (95% UI: 113449-840002) in 2021 (**Figure 1**).

**Figure 1** demonstrates progressive increases in ASMR, ASDR, and ASYR for high BMI-attributable OC from 1990-2021. Specifically, ASMR/100000 rose from 0.32 (95% UI: 0.07-0.61) in 1990 to 0.38 (95% UI: 0.09-0.67) in 2021, with EAPC 1.03 (95% CI: 0.85-1.21) (**Table 1**). Concurrently, ASDR/100000 increased from 8.72 (95% UI: 1.78-16.41) to 10.56 (95% UI: 2.5-18.57) over this period, yielding EAPC 1.09 (95% CI: 0.93-1.25) (**Table 2**). The Figure further illustrates YLLs, YLDs, and corresponding ASR trends.

**Table 1:** Deaths and ASMR for ovarian cancer attributable to high BMI among WCBA from 1990 to 2021.

Year	1990		2021		EAPC (95% *CI*)
	Number	ASR (per 100,000)	Number	ASR (per 100,000)	
Global	6850 (1423-12865)	0.32 (0.07-0.61)	17344 (4141-30810)	0.38 (0.09-0.67)	1.03 (0.85-1.21)
Age					
20-24	6 (-3-16)	0 (0-0.01)	31 (4-59)	0.01 (0-0.02)	4.55 (4.34-4.75)
25-29	19 (0-40)	0.01 (0-0.02)	67 (12-128)	0.02 (0-0.04)	3.46 (3.31-3.61)
30-34	45 (5-90)	0.02 (0-0.05)	138 (29-253)	0.05 (0.01-0.08)	2.36 (2.24-2.48)
35-39	96 (14-186)	0.06 (0.01-0.11)	264 (60-477)	0.1 (0.02-0.17)	1.63 (1.47-1.78)
40-44	202 (35-388)	0.14 (0.03-0.28)	527 (122-944)	0.21 (0.05-0.38)	0.99 (0.85-1.14)
45-49	361 (69-676)	0.32 (0.06-0.59)	995 (234-1767)	0.42 (0.1-0.75)	0.64 (0.48-0.79)
SDI region					
Low SDI	62 (0-139)	0.05 (0-0.11)	433 (68-840)	0.15 (0.02-0.29)	3.61 (3.52-3.7)
Low-middle SDI	187 (13-392)	0.06 (0-0.12)	1771 (357-3220)	0.22 (0.04-0.41)	4.69 (4.49-4.89)
Middle SDI	543 (65-1064)	0.1 (0.01-0.19)	3833 (904-6972)	0.26 (0.06-0.48)	3.16 (3.09-3.24)
High-middle SDI	2244 (484-4207)	0.4 (0.09-0.75)	5095 (1241-9060)	0.48 (0.12-0.85)	0.47 (0.37-0.58)
High SDI	3802 (812-7120)	0.61 (0.13-1.15)	6187 (1531-10979)	0.57 (0.14-1.01)	-0.36 (-0.49–0.23)

**Table 2:** DALYs and ASDR for ovarian cancer attributable to high BMI among WCBA from 1990 to 2021.

Year	1990		2021		EAPC (95% *CI*)
	Number	ASR (per 100,000)	Number	ASR (per 100,000)	
Global	188874 (38401-355691)	8.72 (1.78-16.41)	477248 (113449-840002)	10.56 (2.5-18.57)	1.09 (0.93-1.25)
Age					
20-24	428 (-213-1142)	0.18 (-0.09-0.47)	2182 (291-4194)	0.74 (0.1-1.43)	4.56 (4.35-4.77)
25-29	1227 (15-2643)	0.56 (0.01-1.2)	4413 (818-8377)	1.52 (0.28-2.88)	3.48 (3.32-3.63)
30-34	2714 (278-5417)	1.43 (0.15-2.85)	8328 (1763-15166)	2.79 (0.59-5.07)	2.37 (2.25-2.49)
35-39	5255 (780-10199)	3.03 (0.45-5.88)	14528 (3287-26276)	5.23 (1.18-9.46)	1.64 (1.49-1.79)
40-44	10048 (1760-19196)	7.17 (1.26-13.69)	26242 (6096-46794)	10.58 (2.46-18.86)	1 (0.86-1.15)
45-49	15974 (3026-29871)	14.04 (2.66-26.25)	44223 (10412-78821)	18.77 (4.42-33.45)	0.64 (0.49-0.8)
SDI region					
Low SDI	2127 (24-4781)	1.6 (0.01-3.56)	14943 (2349-28748)	4.68 (0.74-9.04)	3.55 (3.45-3.65)
Low-middle SDI	6283 (459-13117)	1.78 (0.13-3.74)	57906 (11973-104671)	6.96 (1.43-12.58)	4.69 (4.49-4.89)
Middle SDI	17976 (2083-35888)	2.99 (0.36-5.9)	121139 (28727-221614)	8.25 (1.95-15.09)	3.2 (3.13-3.26)
High-middle SDI	65389 (13956-121986)	11.71 (2.49-21.84)	138126 (33461-244871)	13.54 (3.26-24.13)	0.33 (0.25-0.42)
High SDI	96742 (20587-180828)	16.78 (3.57-31.36)	144449 (36080-255583)	15.13 (3.79-26.82)	-0.43 (-0.56–0.3)

### Age-specific disease burden of OC attributable to high BMI from 1990 to 2021

**Figure 2:**
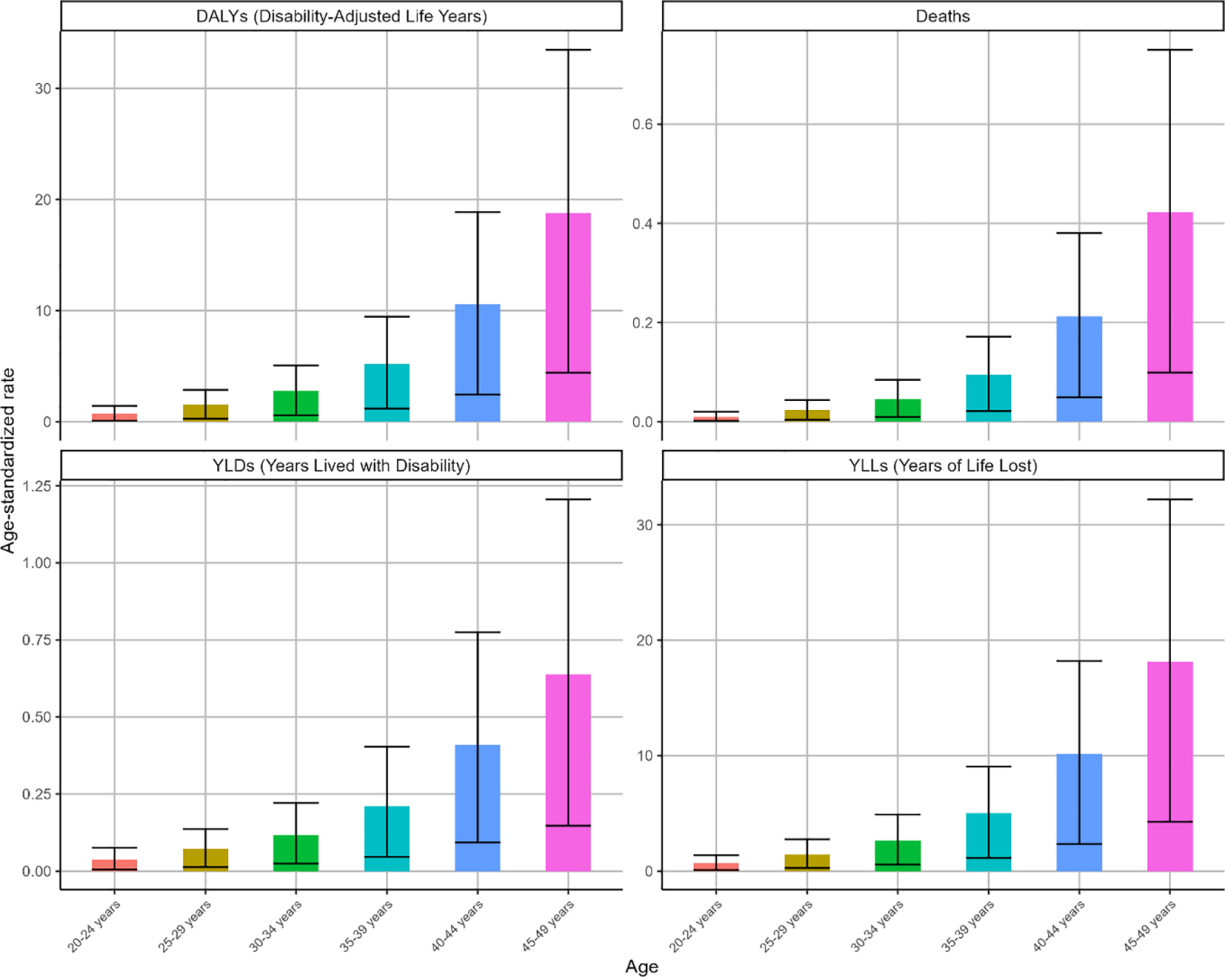
The ASR of deaths, DALYs, YLDs and YLLs for OC attributable to high BMI by age in 2021. Indicators: age-specific ASMR and ASDR (per 100,000), with corresponding deaths and DALYs counts.

Globally, both deaths and DALYs for high BMI-attributable OC demonstrated age-dependent escalation in 2021 (**Supplementary Figure S1**), peaking within the 45–49 age cohort. Parallel trends were observed in ASMR and ASDR metrics. This age group recorded 995 deaths (95% UI: 234-1767) and 44223 DALYs (95% UI: 10412-78821), with an ASMR of 0.42 (95% UI: 0.1-0.75) and an ASDR of 18.77 (95% UI: 4.42-33.45) (**Figure 2**).

**Figure 3:**
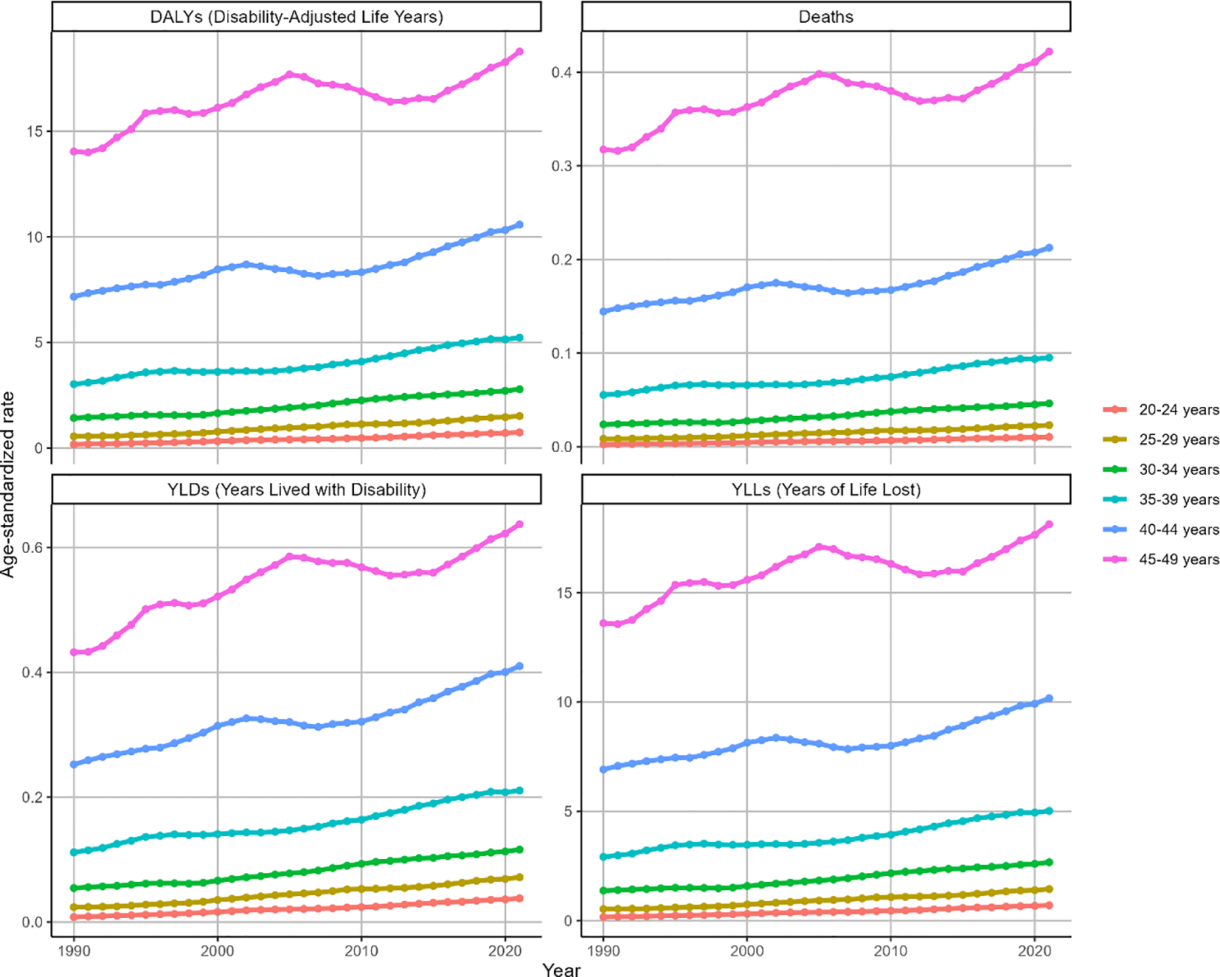
Trends in the ASR of deaths, DALYs, YLDs and YLLs for OC attributable to high BMI by age from 1990 to 2021. Trends shown for ASMR, ASDR, ASYR (per 100,000) and deaths, DALYs, YLDs, YLLs counts across age groups.

Globally, ASMR and ASDR for high BMI-attributable OC exhibited upward trajectories across all age groups during the observation period (**Figure 3**). Notably, while other cohorts demonstrated gradual increases, the 45–49 age group displayed distinct fluctuations: an initial ascent (1990-2005), subsequent decline (2005-2012), and marked resurgence (2012-2021). Overall, this cohort maintained upward trends in ASMR (EAPC = 0.64, 95% CI: 0.48-0.79) and ASDR (EAPC = 0.64, 95% CI: 0.49-0.8) as shown in **Figure 3**. Corresponding changes in deaths, DALYs, YLDs, and YLLs are presented in **Supplementary Figure S2**.

### Disease burden of OC attributable to high BMI in different SDI regions and countries from 1990 to 2021

In 2021, low SDI regions recorded the minimal ASMR/100000 for BMI-attributable OC (0.15, 95% UI: 0.02-0.29), while high SDI regions exhibited the peak value (0.57, 95% UI: 0.14-1.01). A similar trend was seen for the ASDR (per 100,000) [low SDI regions (4.68, 95% UI: 0.74-9.04); high SDI region (15.13, 95% UI: 3.79-26.82)]. ASMR and ASDR for ovarian cancer demonstrated positive correlations with rising SDI levels globally (**Supplementary Figure S3**). Corresponding deaths and DALYs metrics are illustrated in **Supplementary Figure S4**.

**Figure 4:**
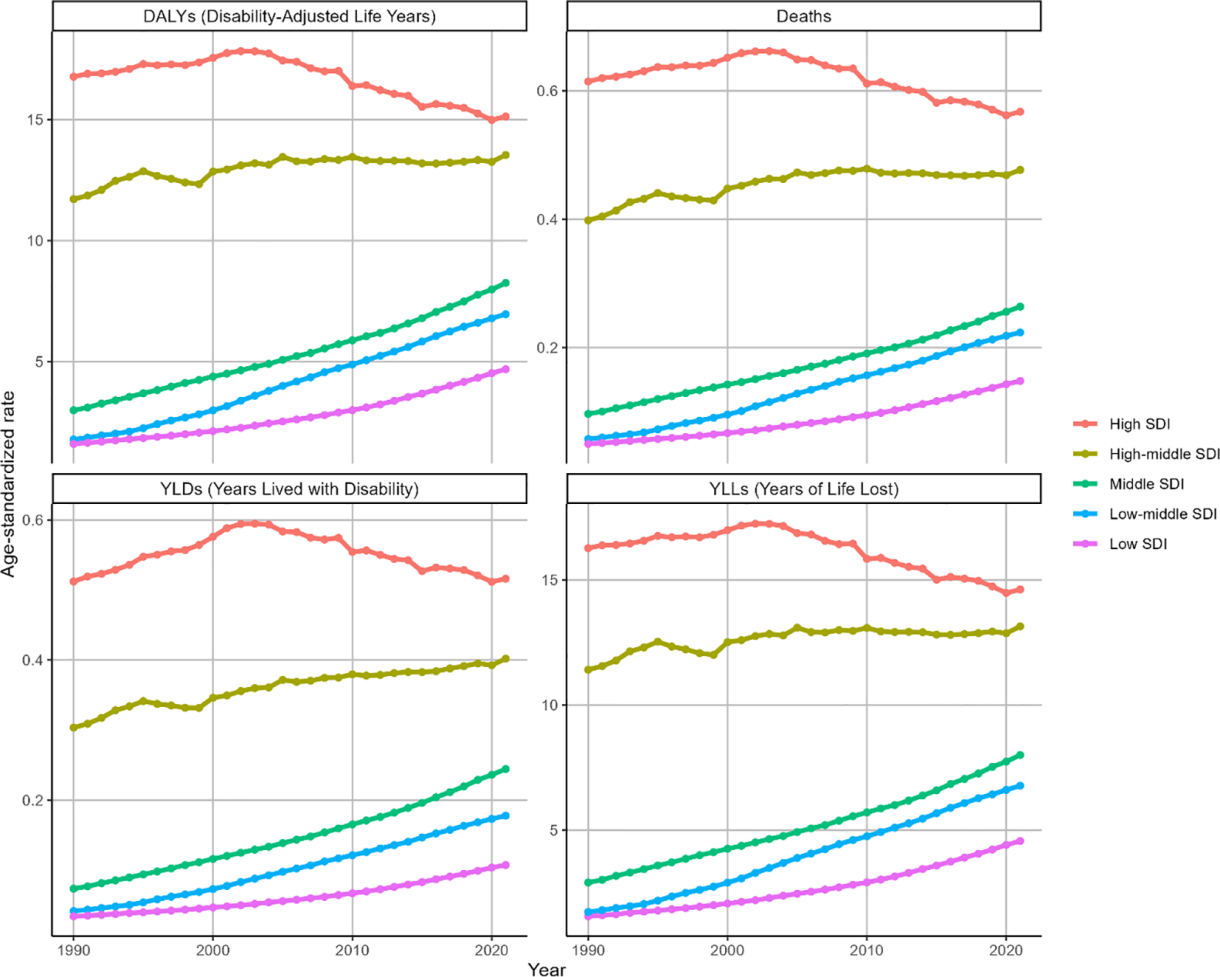
Trends in the ASR of deaths, DALYs, YLDs and YLLs for OC attributable to high BMI by SDI region from 1990 to 2021. Indicators: ASMR, ASDR, ASYR by SDI region (per 100,000), with matching deaths and DALYs counts.

During 1990-2021, ASMR for BMI-attributable OC exhibited significant upward trends across multiple SDI regions: low SDI (EAPC = 3.61, 95% CI: 3.52-3.7), lower-middle SDI (EAPC = 4.69, 95% CI: 4.49-4.89), middle SDI (EAPC = 3.16, 95% CI: 3.09-3.24), and high-middle SDI (EAPC = 0.47, 95% CI: 0.37-0.58). Notably, the high-middle SDI region demonstrated a comparatively attenuated increase. Conversely, high SDI regions displayed distinct patterns: gradual ASMR elevation from 1990 to 2003 (peak: 0.66, 95% UI: 0.16-1.21), followed by marked decline through 2021, culminating in an overall downward trajectory (EAPC=-0.36, 95% CI:-0.49–0.23) as depicted in **Figure 4**. Parallel ASDR trends are documented in **Figure 4** and **Table 2**.

**Figure 5:**
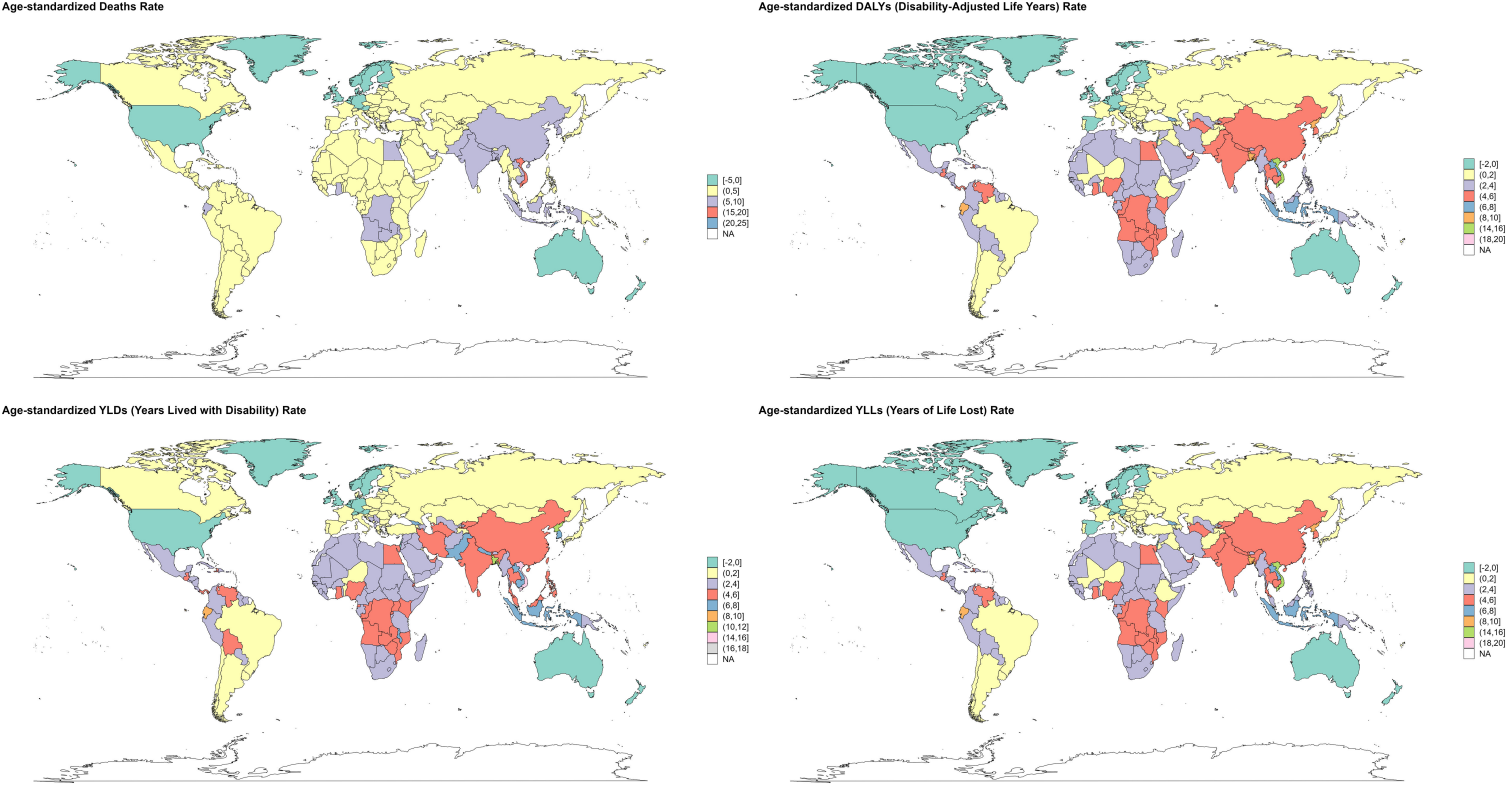
World map of EAPC for OC attributable to high BMI from 1990 to 2021. Choropleth maps report ASMR and ASDR (per 100,000) and EAPC as specified; to facilitate cross-figure comparison, color-scale categories are harmonized across all world maps (see also **Supplementary Figures S6**, **S7**).

In 2021, the highest ASMR for BMI-attributable OC occurred in United Arab Emirates (3.73, 95% UI: 1.05-6.55), Bahrain (1.35, 95% UI: 0.41-2.54), and Qatar (1.23, 95% UI: 0.38-2.27). Correspondingly, the top ASDR values were documented in United Arab Emirates (80.08, 95% UI: 22.77-140.04), Bahrain (36.75, 95% UI: 11.28-67.96), and Bahamas (35.80, 95% UI: 9.60-65.14) (**Supplementary Figure S7**). National variations in deaths, DALYs, YLDs, and YLLs are visualized in **Supplementary Figure S6**. During 1990-2021, ASMR declined in 12 nations but increased in 192, with Ecuador (EAPC = 9.36, 95% CI: 7.96-10.78), Republic of Korea (EAPC = 7.77, 95% CI: 7.23-8.31), and Indonesia (EAPC = 7.28, 95% CI: 6.86-7.69) showing the most rapid ascents. Similarly, ASDR decreased in 17 countries while rising in 187, where Ecuador (EAPC = 9.1, 95% CI: 7.7-10.52), Republic of Korea (EAPC = 7.34, 95% CI: 6.85-7.83), and Indonesia (EAPC = 6.97, 95% CI: 6.57-7.37) exhibited the steepest increases (**Figure 5**).

### Projections to 2050 on the burden of OC attributable to high BMI

**Figure 6:**
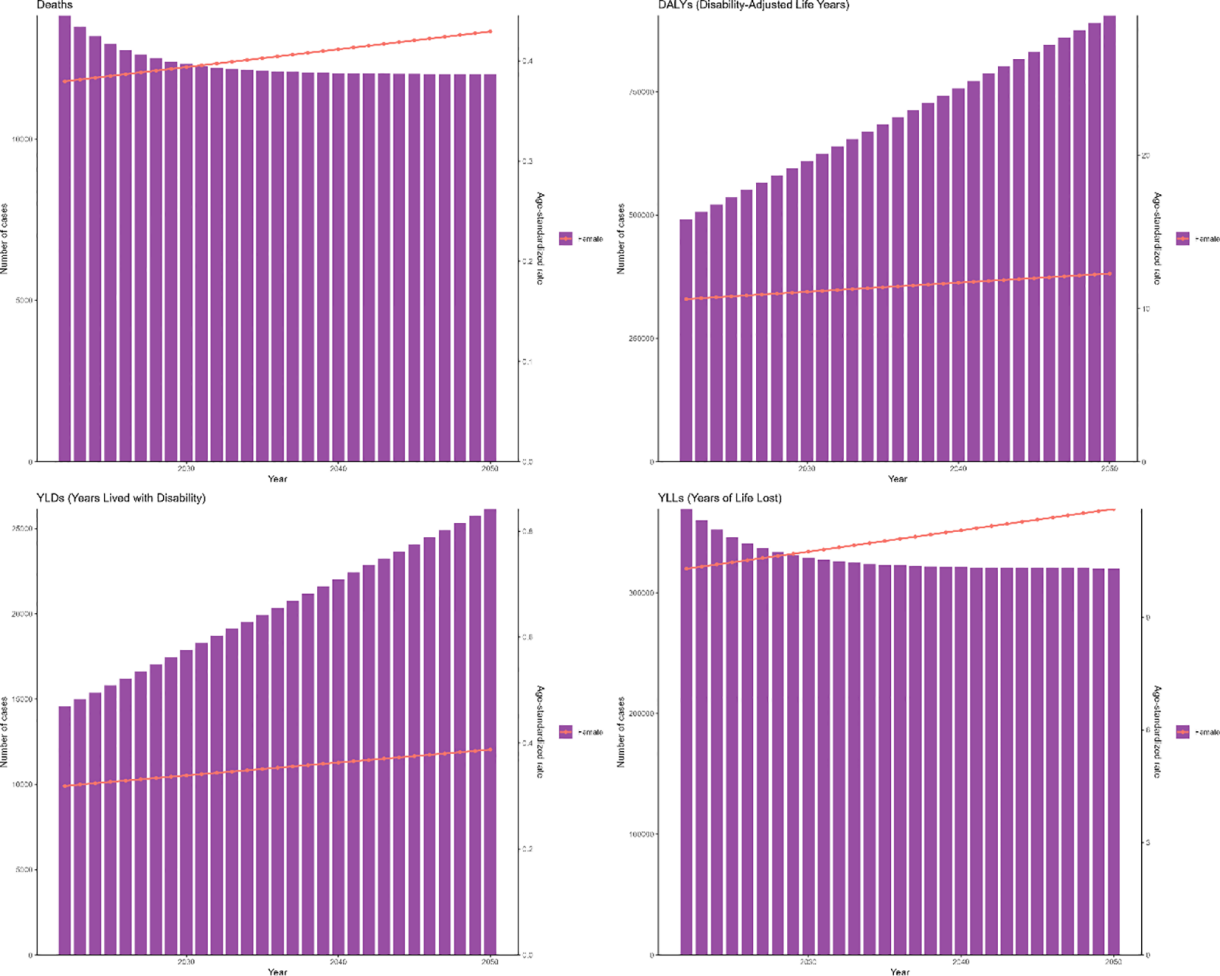
The projection of the burden for OC attributable to high BMI from 2022 to 2050 worldwide by using ARIMA model. Projections shown for ASMR, ASDR and other outcomes where applicable; point forecasts are presented alongside 95% prediction intervals.

ARIMA model projections indicate a steady rise in ASMR for BMI-attributable OC from 2022-2050, reaching 0.43 (95% HDI: 0.40–0.46) by 2050. Conversely, obesity-attributable OC deaths are projected to decline during this period. The ASR of YLLs parallels ASMR trajectories, while DALYs demonstrate significant elevation from 2022 onward. ASDR shows moderate but consistent growth, attaining 12.28 (95% HDI: 11.58-12.98) by 2050, with ASYR exhibiting analogous trends as depicted in **Figure 6**.

ES model projections forecast a moderate yet consistent rise in BMI-attributable OC ASMR, reaching 0.38 (95% HDI: 0.35–0.41) by 2050. Conversely, obesity-driven OC deaths are projected to decline at rates marginally exceeding ARIMA model predictions. ASR of YLLs closely parallels ASMR trajectories, while DALYs demonstrate substantial elevation during 2022-2050. ASDR exhibits gradual growth, attaining 11.16 (95% HDI: 9.36-12.95) by 2050, with ASYR showing analogous progression as illustrated in **Supplementary Figure S8**.

## Discussion

Employing the GBD framework, we quantify global OC burdens associated with excessive BMI in 15–49 year females across 1990-2021, with modeling extending to 2050. This investigation reveals an intensifying burden disproportionately affecting high and upper-middle SDI territories. The evidence mandates tailored interventions targeting escalating OC rates and deaths attributable to clinically significant obesity.

Attributable to elevated BMI, worldwide OC burden has demonstrated substantial growth since 1990, revealing pronounced interregional divergences. Distinct disease burden gradients are observed, with North American and Western European high-SDI populations demonstrating significantly greater age-standardized incidence and mortality versus low/low-middle SDI counterparts (15, 16). This trend aligns with established epidemiological patterns linking socioeconomic development to obesity-related cancers, where sedentary lifestyles and calorie-dense diets drive metabolic dysregulation (17, 18). Conversely, low-SDI regions demonstrate a concerning upward trajectory in BMI-attributable OC burden despite lower baseline rates, emphasizing the urgency of implementing cost-effective screening programs in resource-limited settings (19).

Age-specific analyses reveal peak disease burden in the 45–49 age group, consistent with ovarian cancer’s propensity for late-stage presentation (20). This finding supports intensified screening protocols (e.g., transvaginal ultrasound with CA-125 testing) and lifestyle counseling for perimenopausal women (21). Younger WCBA populations may benefit from preventive education on weight management, while postmenopausal women require enhanced surveillance given diagnostic challenges in this demographic (22).

Significantly, this analysis identified diverging future trajectories: a continuing upward trajectory in OC incidence contrasted with progressive mortality burden reduction. The documented mortality reduction likely reflects refinements in cytoreductive surgery protocols, particularly the adoption of intraoperative tumor mapping and R0 resection criteria, which collectively enhance therapeutically meaningful cytoreduction outcomes (23). Bevacizumab and PARP inhibitors (olaparib, niraparib) exhibit therapeutic synergy, yielding substantial survival gains in OC through prolonged PFS and OS durations—especially within homologous recombination-deficient cohorts where VEGF blockade augments DNA repair pathway suppression (24, 25). Emerging immune checkpoint modulators (including PD-1/PD-L1 antagonists) in active clinical development show potential to extend survival outcomes alongside platinum-taxane chemotherapy regimens (26, 27). Given the globally escalating BMI trends, implementing multifaceted public health interventions becomes imperative. Promoting physical activity, optimizing dietary patterns (e.g., Mediterranean diet adoption), and enhancing accessibility to preventive healthcare services constitute critical strategies for mitigating the future burden of OC (28).

Our findings align with other global burden studies that underscore the escalating trend of obesity-related cancers (29). Empirical evidence indicates marked breast cancer incidence escalation linked to BMI elevation, underscoring metabolic dysregulation’s central contribution to cancer pathogenesis (30). Independent research reveals hyperglycemia-associated OC burden escalation, confirming metabolic dysregulation’s pivotal role in shaping global oncological disease patterns (31). Collectively, these studies highlight the critical need to address metabolic risk factors to effectively mitigate the growing global cancer burden (32).

This study has several limitations that should be considered when interpreting the findings. First, by design, we restricted the analysis to women aged 15–49 years. Although this age range corresponds to the conventional definition of WCBA used in global health reporting and is of particular relevance for reproductive health, most OC cases and deaths occur after age 50. Our results cannot be generalized to the full lifetime burden of OC and should be interpreted as quantifying the high BMI–attributable component of OC burden among pre- and perimenopausal women, complementing all-age GBD analyses (10, 29). Second, because GBD 2021 provides cause-level OC estimates without histologic or menopausal stratification, we were unable to investigate the known heterogeneity of adiposity–OC associations across histologic subtypes and menopausal status (33). Third, high BMI was the only adiposity indicator consistently available in the GBD comparative risk assessment; more refined measures of body composition and fat distribution have been shown to be stronger predictors of cancer outcomes but are not yet available at the global time-series scale required for our approach (34, 35). Fourth, we focused on high BMI as a single risk factor and did not formally model the joint or mediating effects of other metabolic and lifestyle exposures (e.g., high fasting glucose, physical inactivity, diet) and health-system determinants. Recent cluster-based analyses of gynecologic cancers and large-scale machine learning–Mendelian randomization frameworks illustrate how multifactorial, data-driven approaches can extend beyond the single-risk-factor design used here (36, 37). Our projections should therefore be interpreted as a conservative, BMI-specific component of the future OC burden among WCBA rather than a comprehensive model of all contributing determinants.

## Conclusion

Collectively, this investigation delineates a progressively intensifying worldwide OC burden mediated by elevated BMI in WCBA. These findings necessitate precision-based interventions disproportionately targeting high-SDI settings, aiming to counteract expanding adiposity epidemics and their oncological impact. By informing evidence-based policies and advocating for lifestyle modifications, we can strive to diminish the future impact of this devastating disease. Subsequent research must prioritize delineating adiposity-oncogenesis pathways and formulating risk-tailored preventive frameworks to optimize global female health trajectories.

## Data Availability

The original contributions presented in the study are included in the article/**Supplementary Material**. Further inquiries can be directed to the corresponding authors.
