# Supplementary material for: Global burden of ovarian cancer attributable to high body mass index among women of childbearing age from 1990 to 2021 and projections to 2050: a systematic analysis for the global burden of disease study 2021
Source: Front Oncol. 2025 Dec 18;15:1695717. doi: 10.3389/fonc.2025.1695717 (PMC12756064; doi:10.3389/fonc.2025.1695717)
Supplement: Supplementary file 1 [file DataSheet1.docx]

**Supplementary materials**

**
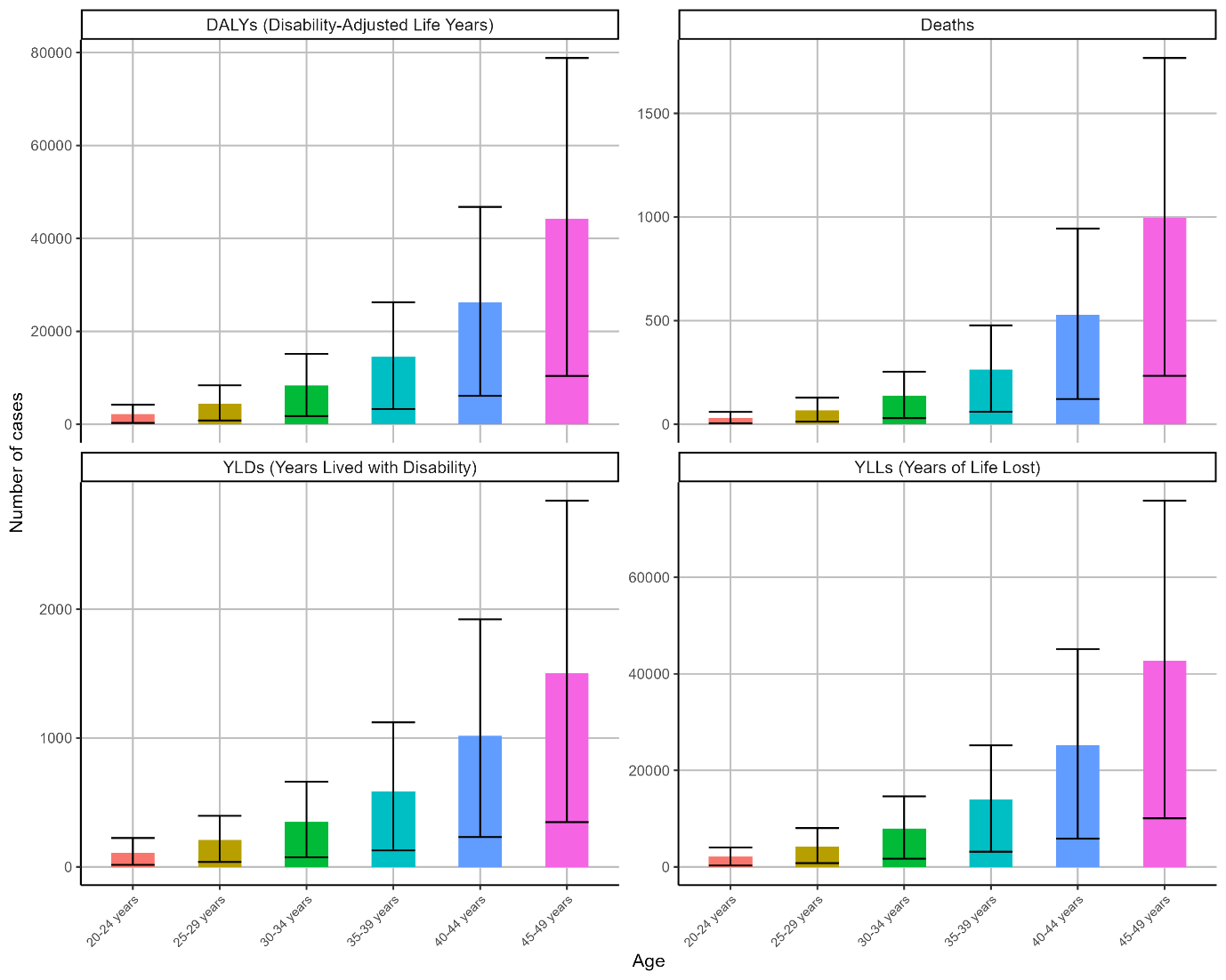
**

**Figure S1.** The deaths, DALYs, YLDs and YLLs due to OC attributable to high BMI by age in 2021. Indicators and units: deaths and DALYs counts; ASMR, ASDR, ASYR per 100,000; YLDs and YLLs counts.


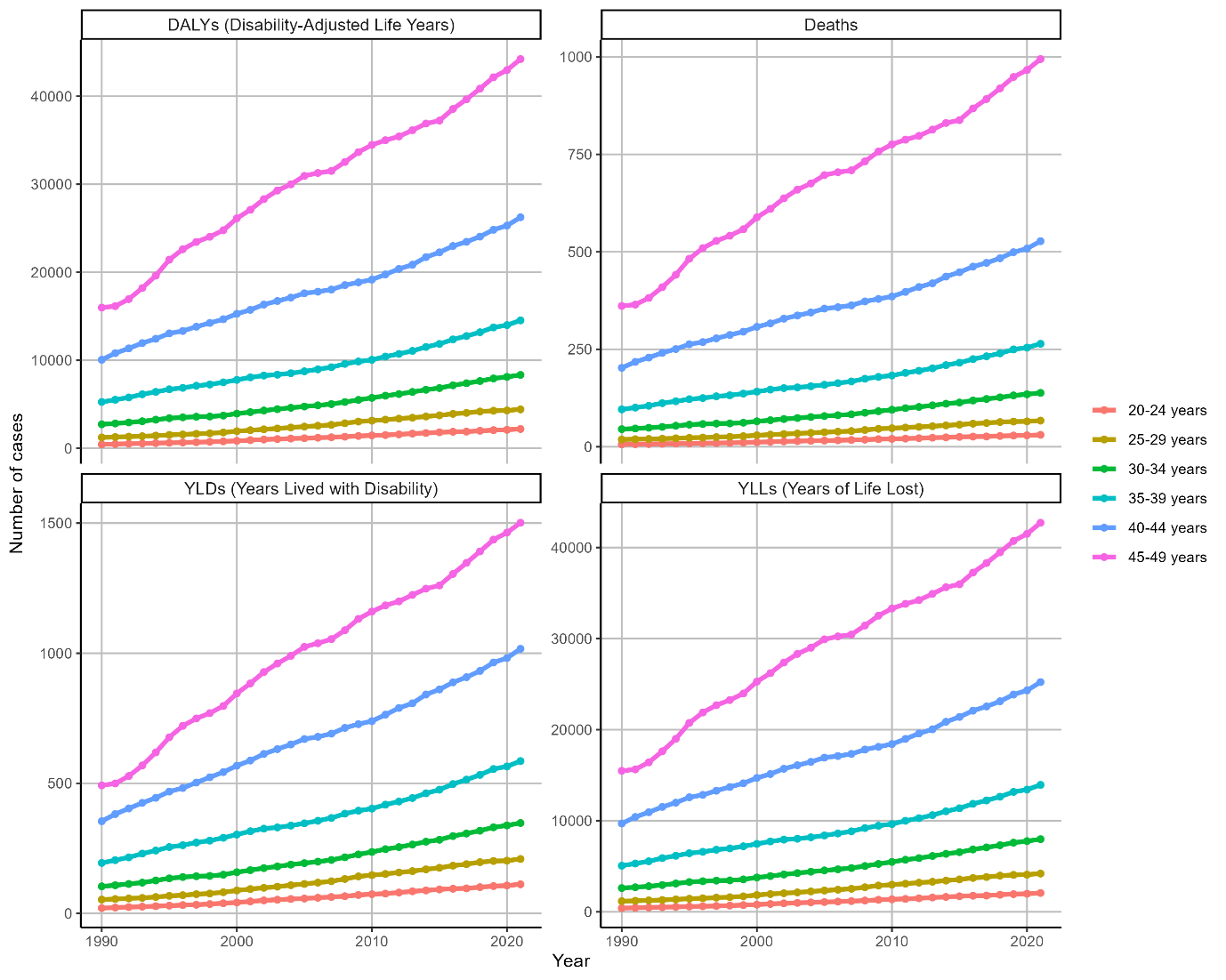


**Figure S2.** The trends for deaths, DALYs, YLDs and YLLs due to OC attributable to high BMI by age from 1990 to 2021. Trends for ASMR, ASDR, ASYR (per 100,000) and deaths, DALYs, YLDs, YLLs counts by age group.


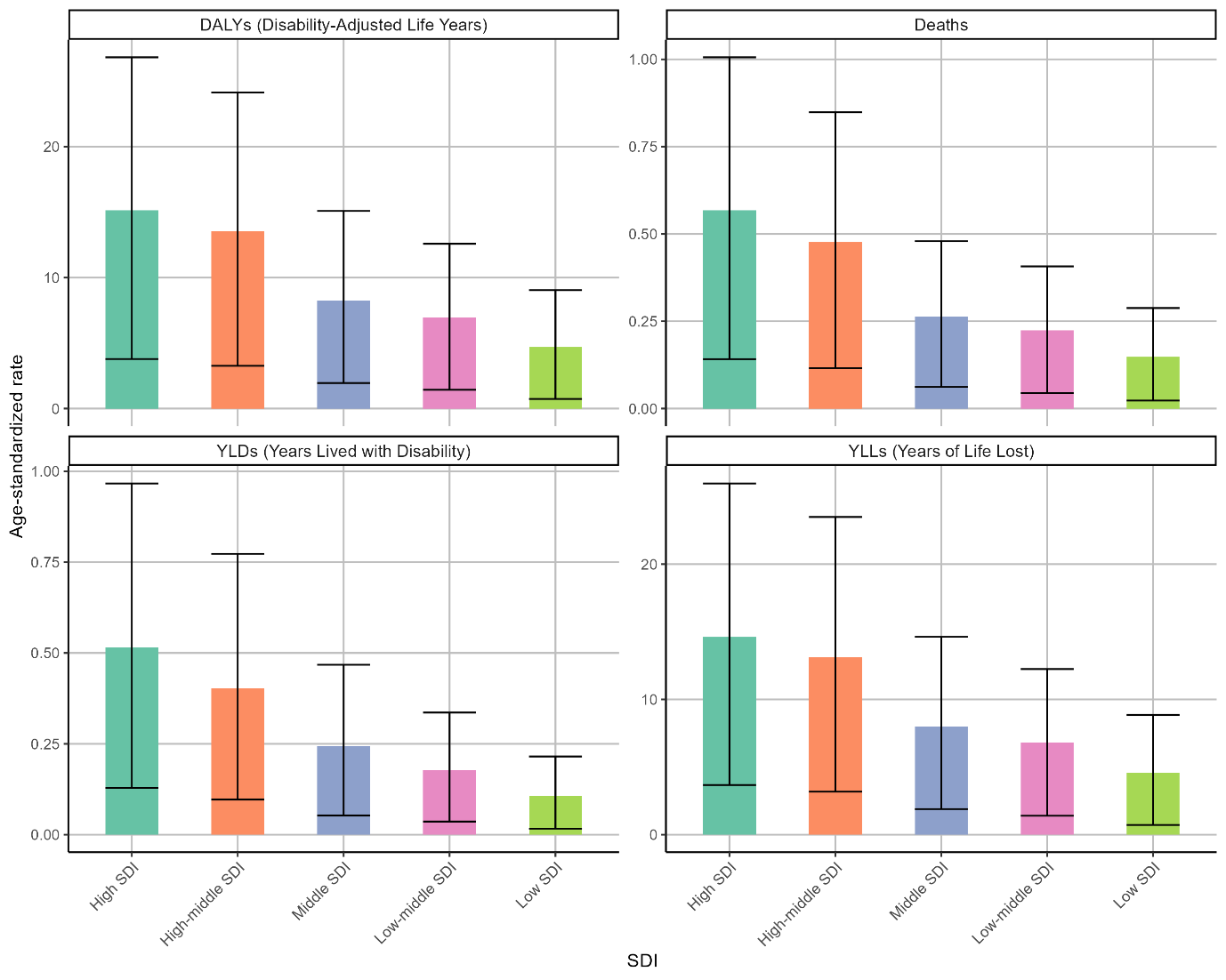


**Figure S3.** **Age-standardized rates for OC attributable to high BMI by SDI region in 2021.** Units: **ASMR**, **ASDR**, **ASYR** per **100,000**.


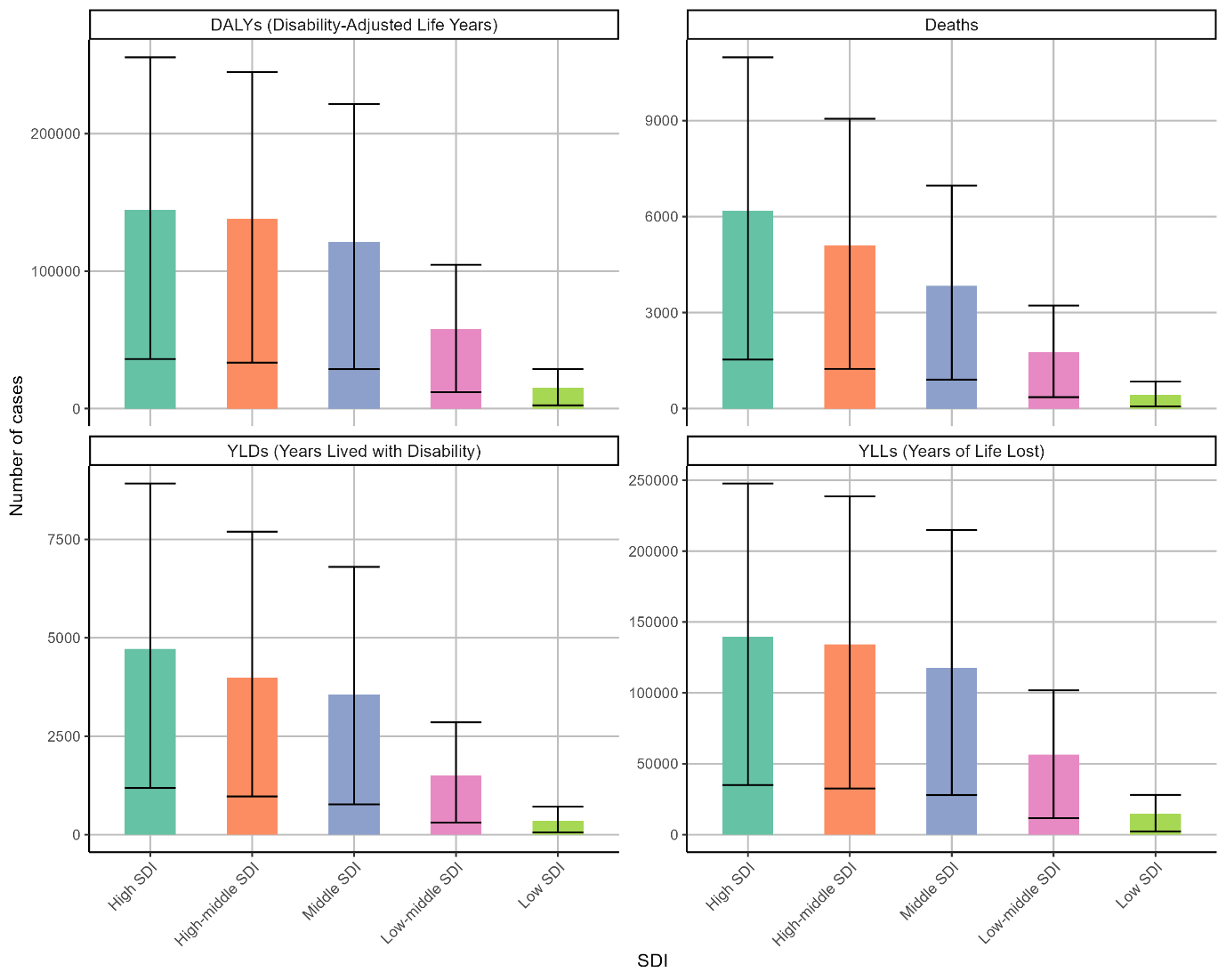


**Figure S4.** **Deaths and DALYs due to OC attributable to high BMI by SDI region in 2021.** (Corresponding rates are reported elsewhere as **ASMR/ASDR** per **100,000**.)


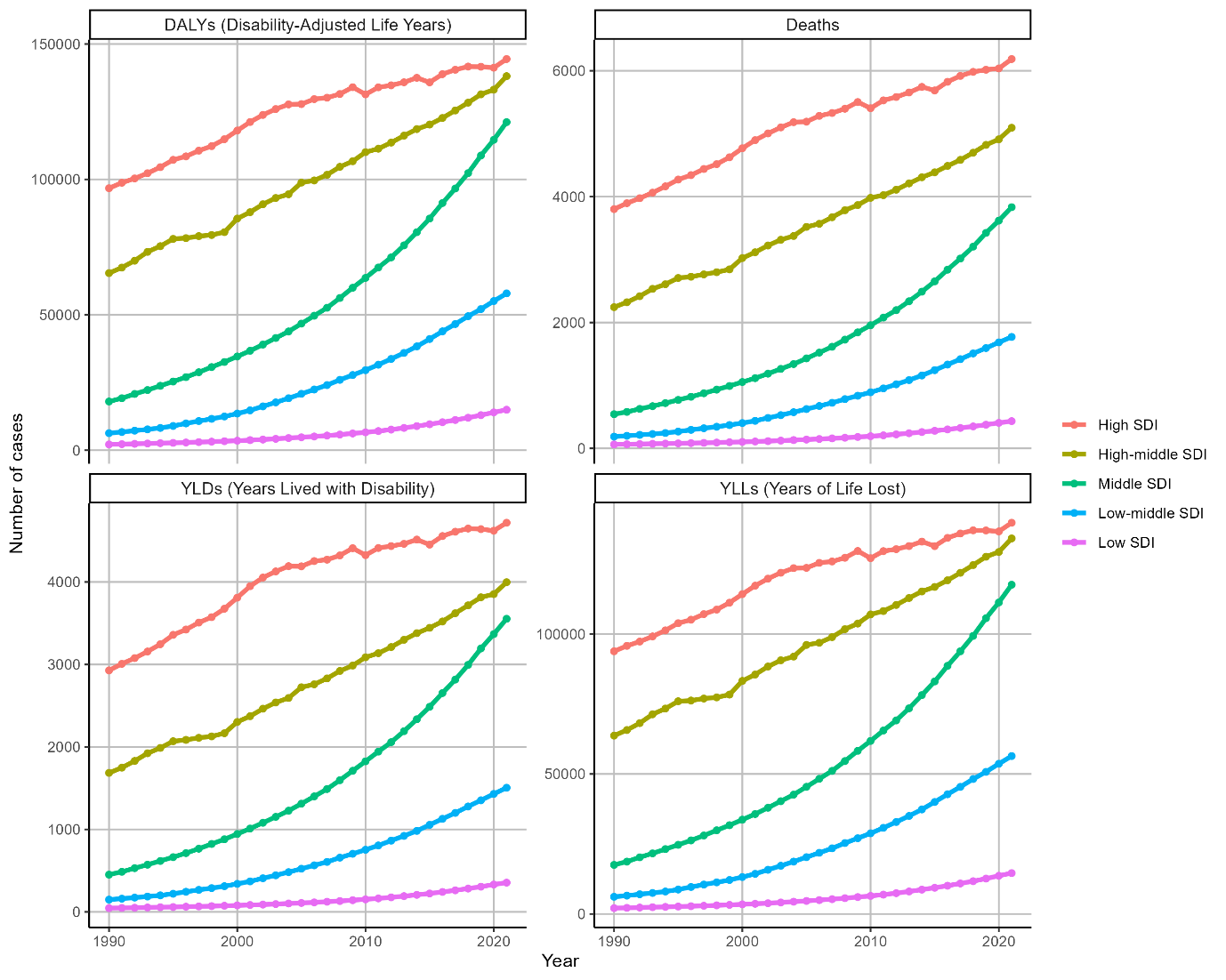


**Figure S5.** **Trends by SDI region for OC attributable to high BMI, 1990–2021.** Trends shown for rates (**ASMR**, **ASDR**, **ASYR** per **100,000**) and counts (deaths, DALYs, YLDs, YLLs).


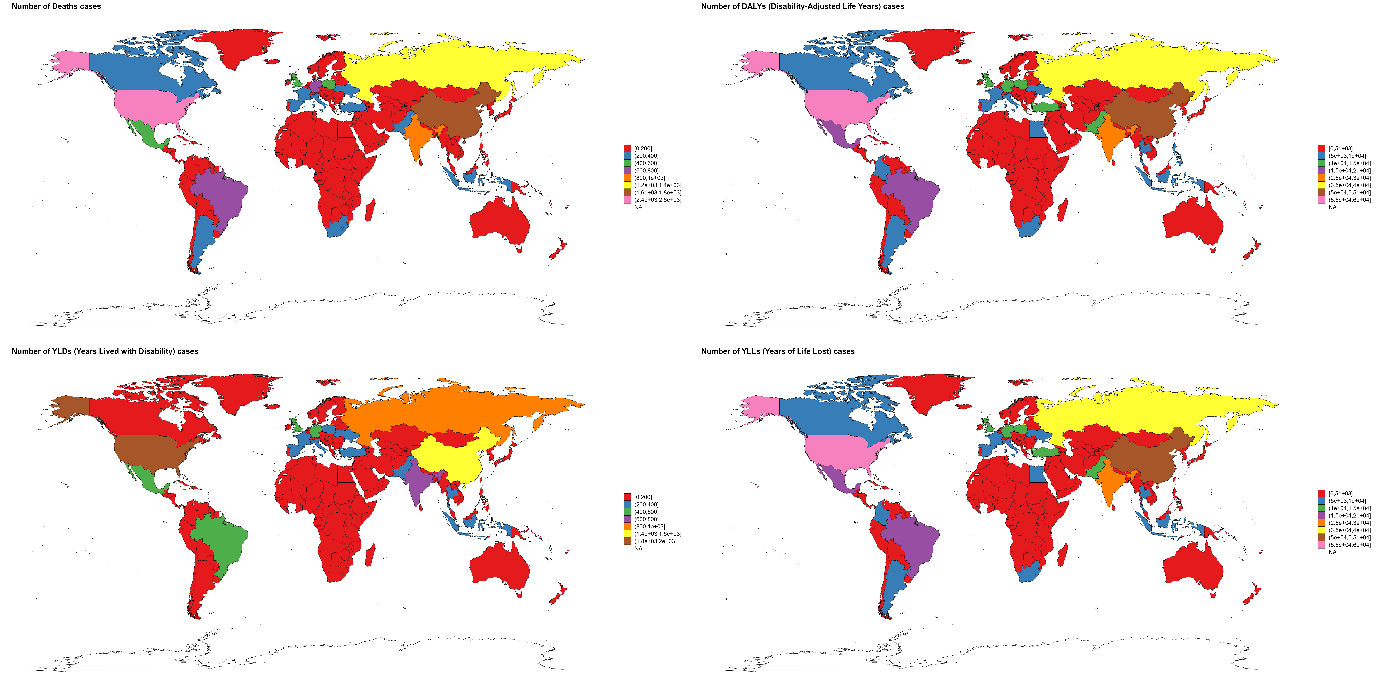


**Figure S6.** World map of deaths, DALYs, YLDs and YLLs due to OC attributable to high BMI in 2021. Choropleth map; units are counts. Color-scale categories are standardized across S6 and S7 to aid visual comparability.


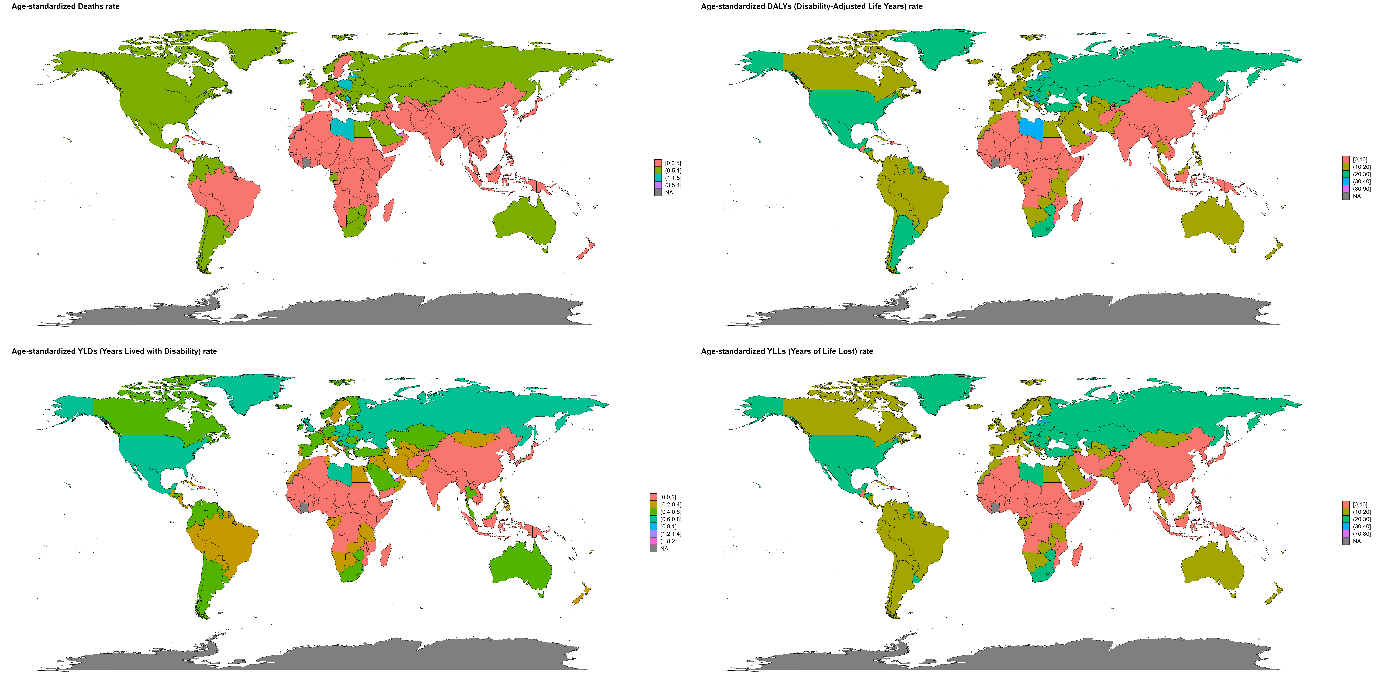


**Figure S7.** World map of age-standardized rates (ASMR, ASDR, ASYR) for OC attributable to high BMI in 2021. Units: per 100,000. Color-scale categories match those used in Figure S6 and the main-text Figure 5.


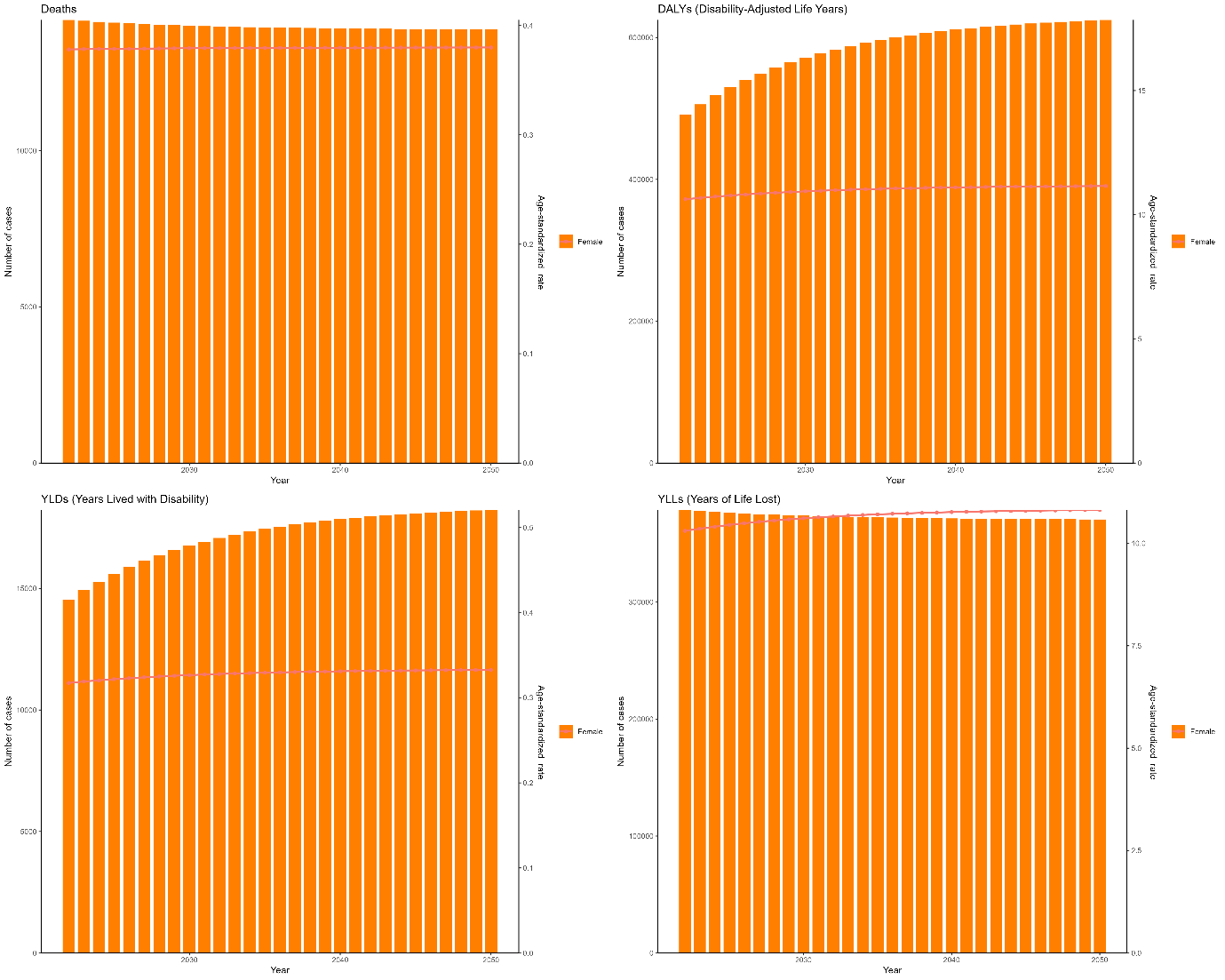


**Figure S8.** The projection of the burden for OC attributable to high BMI from 2022 to 2050 worldwide by using ES model. Projections shown as point forecasts with 95% prediction intervals where applicable.
